# Quality of Patient-Centered Care Provided to Patients Attending Hematological Cancer Treatment Centers

**DOI:** 10.3390/ijerph15030549

**Published:** 2018-03-19

**Authors:** Flora Tzelepis, Tara Clinton-McHarg, Christine L Paul, Robert W Sanson-Fisher, Douglas Joshua, Mariko L Carey

**Affiliations:** 1 Priority Research Centre for Health Behaviour, University of Newcastle, Callaghan, NSW 2308, Australia; Tara.Clinton-McHarg@newcastle.edu.au (T.C.-M.); Chris.Paul@newcastle.edu.au (C.L.P.); Rob.Sanson-Fisher@newcastle.edu.au (R.W.S.-F.); Mariko.Carey@newcastle.edu.au (M.L.C.); 2 Hunter Medical Research Institute, New Lambton Heights, NSW 2305, Australia; 3 Institute of Haematology, New South Wales Pathology, Royal Prince Alfred Hospital, University of Sydney, Sydney, NSW 2006, Australia; douglas.joshua@sydney.edu.au

**Keywords:** patient-centered, quality of care, hematology, cancer

## Abstract

The Institute of Medicine has recommended that improvements are needed in patient-centered care. This study examined hematological cancer patients’ perceptions of which aspects of cancer care were being delivered well and areas that required improvement, and whether patient characteristics, or the treatment center they attended, were associated with quality of patient-centered care. Participants were recruited via three Australian hematological cancer treatment centers and completed a paper-and-pen survey assessing sociodemographic, disease, and psychological and treatment characteristics at recruitment. A second survey that contained the Quality of Patient-Centered Cancer Care measure was completed one month after recruitment (*n* = 215). The most frequently delivered feature of patient-centered cancer care was hospital staff showing respect for patients (91.0%). The area of care reported most commonly as not being delivered was hospital staff helping the patient find other cancer patients to talk to (29.8%). Patients without depression reported higher perceived quality of treatment decision-making, co-ordinated and integrated care, emotional support, follow-up care, respectful communication, and cancer information than patients with depression. The treatment center that was attended was associated only with the quality of cancer information patients received. Privacy issues may hinder staff connecting patients directly but this could be overcome via referrals to cancer organizations that offer peer support services.

## 1. Introduction

Hematological cancers include leukemias, lymphomas, and myeloma and account for 6.5% of cancers globally [1]. Treatments for hematological cancers include chemotherapy, radiotherapy, and bone marrow transplantation [2], and patients may experience extended recovery trajectories [3]. For instance, Australian data has shown that acute myeloid leukemia patients have a longer average hospital stay than any other cancer type [3]. Given that hematological cancer patients may have regular and prolonged interaction with the health care system, assessing their perceptions about the care they received is important for prioritizing quality improvement initiatives.

The Institute of Medicine (IOM) has suggested that in order to achieve high quality health care, improvements are needed across the areas of safety, effectiveness, timeliness, efficiency, equity, and patient-centeredness [4]. The IOM has endorsed six dimensions of patient-centeredness proposed by Gerteis et al. [5] that stipulate that care must (1) be respectful to patients’ values, preferences, and expressed needs; (2) be coordinated and integrated; (3) provide information, communication, and education; (4) ensure physical comfort; (5) provide emotional support to relieve fear and anxiety; and (6) involve family and friends [4].

To accurately assess the provision of patient-centered care, comprehensive and psychometrically robust patient-reported measures are needed [6] and may include measures of satisfaction with care [7] or experiences with care [8]. Such measures are required because only the patient can determine whether care aligns with their values, preferences, and needs, and if they received the level of information desired [6]. The Picker Patient Experience Questionnaire has been widely administered in Europe and assesses information and education, co-ordination of care, physical comfort, emotional support, respect for patient preferences, involvement of family and friends, continuity and transition, and overall impression [9,10]. The Consumer Assessment of Healthcare Providers and Systems (CAHPS) Hospital survey is widely used in the USA and assesses communication with doctors, communication with nurses, communication about medication, nursing services, physical environment, pain control, and discharge information [11]. However, to the authors’ knowledge there is no evaluation of the psychometric properties of the Picker Patient Experience Questionnaire or CAHPS Hospital survey with cancer patients. Furthermore, we conducted a systematic review that found that no existing measure that assessed the quality of patient-centered care in cancer patients met all criteria for adequate validity and reliability [12,13,14,15,16,17,18] and comprehensively addressed all six IOM endorsed dimensions of patient-centered care [19]. Based on the findings of our systematic review [19], we developed the Quality of Patient-Centered Cancer Care (QPCCC) measure that includes items that cover all six IOM-endorsed patient-centered care dimensions and has been found to meet criteria for acceptable face validity, content validity, construct validity, and internal consistency with hematological cancer survivors [20].

Only one previous study has used the QPCCC measure to explore hematological cancer survivors’ perceptions of patient-centered care [21]. This research found that most hematological cancer survivors perceived that staff had been respectful when communicating with them [21]. However, the study also reported that approximately one-third of hematological cancer survivors disagreed that hospital staff helped the patient or their family or friends find others in a similar situation to talk to [21]. This previous study recruited participants via cancer registries and not treatment centers, and no analysis was conducted regarding whether the quality of patient-centered care varied according to the hematological cancer treatment center attended. Other research with hematological cancer survivors has focused on information provision [22,23]. In a Dutch study, 29% of lymphoma and multiple myeloma survivors indicated that they would have liked more information, particularly in relation to the cause and course of disease, late effects of treatment, and psychosocial follow-up [22]. Similarly, a UK study found that lymphoma survivors wanted to discuss the late effects of treatment more than they had done during consultations [23]. These studies focused on information provision and did not assess all of the patient-centered care dimensions endorsed by the IOM.

Limited research has examined the characteristics associated with hematological cancer patients’ perceptions of the quality of patient-centered care. Higher levels of education, younger age, and fewer co-morbid conditions have been associated with higher perceived information provision among hematological cancer survivors [22]. Another study found that factors associated with higher perceived quality of patient-centered care included being employed, younger age, a more recent diagnosis, and a Non-Hodgkin lymphoma diagnosis (compared to leukemia) [21]. In contrast, among hematological cancer survivors, depression or stress were associated with lower perceived quality of patient-centered cancer care [21].

No previous study has used a comprehensive instrument based on all dimensions of patient-centered care (such as the QPCCC) to assess whether the quality of patient-centered care that hematological cancer patients receive varies by treatment center attended. The aims of the current study were to recruit hematological cancer patients from three treatment centers and investigate the following:

(1) perceptions of which aspects of cancer care were commonly delivered and areas that required improved delivery;
(2) the mean quality care scores for each QPCCC subscale; and
(3) whether patient socio-demographic characteristics, cancer history, depression, anxiety, or treatment center attended were associated with perceived patient-centered care.

## 2. Materials and Methods

### 2.1. Setting

Two hematological cancer outpatient treatment centers from New South Wales (centers 2 & 3) and another from Victoria (center 1), Australia participated. Each of the three centers treated at least 300 adult hematological cancer patients per year.

### 2.2. Participants

Patients were eligible to participate if they met the following criteria: (1) confirmed diagnosis of hematological cancer; (2) attending the treatment center for their second or subsequent outpatient appointment (treatment or follow-up) to provide opportunity to reflect on the care they received; (3) aged 18 years and older; (4) physically and mentally able to participate; and (5) able to complete a survey in English.

### 2.3. Procedure

A hematologist or nurse at each hematology treatment center identified potentially eligible patients from the daily clinic list. A research assistant approached potentially eligible patients who were in the clinic waiting room awaiting their appointment. Eligible patients were provided with an information sheet that described the study. Patients who agreed to participate signed a consent form and were provided with a paper-and-pen questionnaire that assessed demographic characteristics, cancer history, treatment information, and anxiety and depression. Patients were asked to complete the questionnaire while in the waiting room and return it to the research assistant. If patients preferred to complete the questionnaire at a later time, they were provided with a reply-paid envelope for the return of the completed survey. If the survey was not returned within two weeks, a reminder survey package was mailed to the patient.

A month after recruitment, patients were mailed a second questionnaire to complete and a reply-paid envelope for the return of the completed survey. This questionnaire contained the QPCCC measure in order to assess the quality of patient-centered cancer care provided. If the survey was not returned within two weeks, a reminder survey package was mailed to the patient. Another reminder survey package was sent to non-respondents after a further four weeks.

All subjects gave their informed consent before they participated in the study. The study was conducted in accordance with the Declaration of Helsinki, and the protocol was approved by the University of Newcastle Human Research Ethics Committee (approval no. H-2010-1324) and ethics committees for each treatment center.

### 2.4. Measures

#### 2.4.1. Quality of Patient-Centered Cancer Care (QPCCC)

The 48-item QPCCC measure examines patients’ perceptions of cancer care across ten factor analytically derived subscales: (1) timely care (4 items, e.g., “I had to wait too long from my first visit with the cancer doctor to getting my cancer diagnosis”); (2) respectful communication (3 items; e.g., “The staff at the hospital showed respect for me”); (3) cancer information (3 items; e.g., “The staff at the hospital gave me information about cancer and treatments to take home (e.g., booklets, websites)”); (4) treatment decision-making (8 items; e.g., “When I was making my most recent treatment decision, doctors at the hospital gave me the time I needed to consider all my treatment options before making a decision”); (5) treatment delivery (7 items; e.g., “During treatment staff at the hospital made sure I did not receive unnecessary tests or treatments”); (6) patient preferences and values (3 items; e.g., “During my treatment, I was able to choose which hospital provided my treatment”); (7) equitable care (2 items; e.g., “The treatment I received at the hospital was too far away from where I lived”); (8) coordinated and integrated care (7 items; e.g., “The staff at the hospital helped me organise transport to and from the hospital”); (9) emotional support (4 items; e.g., “The staff at the hospital helped me deal with being worried, upset or sad”); and (10) follow-up care (5 items; e.g., “After treatment had ended, staff at the hospital explained to me what to expect during follow-up tests”). There are also two single items that examine whether hospital staff assisted the patient to move smoothly (a) back home and (b) between different hospitals, clinics, or health services [20]. The response options are *Strongly agree, Agree, Disagree, Strongly disagree*, and *Not applicable to me*. The QPCCC measure has demonstrated acceptable face validity, content validity, construct validity, and internal consistency (Cronbach’s α = 0.73 to 0.94 for subscales) for hematological cancer survivors [20].

#### 2.4.2. Hospital Anxiety and Depression Scale (HADS)

Anxiety and depression as measured by the 14-item Hospital Anxiety and Depression Scale (HADS) were assessed in the survey completed at recruitment [24]. Scores of 8 or above indicated depression or anxiety on the depression and anxiety subscales, respectively [24]. The HADS has evidence of validity and reliability with cancer populations [25,26].

#### 2.4.3. Other Measures

Details regarding age, sex, home postcode, marital status, education, employment, private health insurance, cancer type, time since diagnosis, and cancer treatments were also collected in the survey completed at recruitment.

### 2.5. Statistical Analysis

Participants who completed the first and second surveys were included in the analyses. Statistical analysis was completed using SAS software version 9.4 (SAS Institute Inc., Cary, NC, USA). Percentages and 95% confidence intervals were used to indicate the areas of cancer care that ≥80% of participants reported were delivered and those that ≥20% of patients indicated were not delivered. Depending on QPCCC item wording, delivered care corresponded to the responses of either ‘strongly agree/agree’ or ‘strongly disagree/disagree’ and vice versa for care not delivered. Means and standard deviations were also calculated for each QPCCC subscale. The subscale scores were calculated by adding all items in the subscale and dividing by the number of non-missing items for participants who answered more than 70% of items in that subscale.

To examine characteristics associated with hematological cancer patients’ perceived quality of care, multiple quantile regression models used each QPCCC subscale score as the outcome and demographic, cancer-related, psychological, or treatment center attended characteristics as independent variables. These multiple quantile regression models adjusted by all these co-variates and we were able to identify whether for each of the QPCCC subscales, perceived quality of care varied by treatment center attended (which was one of the independent variables). Estimates showing the change in median score, with 95% confidence intervals, and adjusted Wald *p*-values were calculated.

## 3. Results

There were 395 eligible patients who were approached to participate, and of these 289 (73.2%) returned the first survey, and 215 (54.4%) completed the second survey distributed one month later that contained the QPCCC measure. The reasons for not participating were not collected from non-participants.

### 3.1. Hematological Cancer Patients’ Characteristics

Table 1 describes participant characteristics. The majority of patients were male (57.7%) and the mean age was 62 years (SD = 13). Most patients were married or living with a partner (73.6%), were not employed (64.0%), lived in an urban location (95.8%), and had private health insurance (60.1%). More than two-fifths of patients had attained a primary school or high school education (43.7%). Participants were diagnosed with lymphoma (43%), leukemia (25.2%), myeloma (24.8%), or other hematological conditions (7%) and approximately half had been diagnosed more than 24 months ago (53.3%). The majority of patients had received chemotherapy (76.4%). Over half the participants attended center 1 (54.5%), 32.9% attended center 2, and 12.7% attended center 3.

Table 2 shows that there were no significant differences in terms of age and gender between those who completed the first survey on patient characteristics and those who did not consent during the initial approach to participate in the study. However, there were statistically significant differences between those who completed the second survey that contained the QPCCC measure and those who completed the first survey only in terms of age (*p* = 0.008), marital status (*p* = 0.003), and employment (*p* = 0.047).

### 3.2. Cancer Care Most Commonly Delivered

There were 12 items, for which ≥80% of patients strongly agreed or agreed had been delivered (Table 3). The most frequently delivered features of cancer care were hospital staff showing respect for them (91.0%), hospital staff talking to patients in a way they could understand (90.8%), and hospital staff showing respect to their family or friends (87.6%). Of the 12 areas reported as being delivered by ≥80% of patients, five related to the QPCCC measure’s treatment delivery subscale, three to respectful communication, two to treatment decision-making, and two to cancer information.

### 3.3. Cancer Care Most Commonly Not Delivered

There were six items, for which ≥20% of participants indicated had not been delivered (Table 4). Patients most frequently “strongly disagreed/disagreed” that hospital staff helped the patient find other cancer patients they could talk to about their cancer experiences (29.8%), hospital staff helped family/friends find others in a similar situation to talk to (28.9%), and doctors at the hospital explained they could get a second medical opinion if they wanted to (26.6%). Three areas of cancer care not received by ≥20% of respondents related to the *coordinated and integrated care* subscale, two to *treatment decision-making*, and one to *patient preferences and values*.

### 3.4. QPCCC Subscale Scores

The mean scores for each QPCCC subscale are described in Table 5. *Respectful communication* (mean = 3.6, SD = 0.6) had the highest mean quality score, while coordinated and integrated care (mean = 2.5, SD = 0.4) had the lowest mean quality score.

### 3.5. Characteristics Associated with Perceived Quality of Care

Table 6 reports the characteristics, including treatment center attended, associated with perceived quality of care.

#### 3.5.1. Treatment Delivery

After adjusting for all co-variates, compared to those diagnosed with leukemia, a lymphoma diagnosis was associated with higher perceived quality of treatment delivery. A high school education or below and no private health insurance were also associated with higher perceived quality of treatment delivery.

#### 3.5.2. Treatment Decision-Making

Compared to those diagnosed with leukemia, a lymphoma diagnosis was associated with higher perceived quality of treatment decision-making after adjusting for all co-variates. A high school education or below and not having depression were also associated with higher perceived quality of treatment decision-making. Not being anxious was associated with lower perceived quality of treatment decision-making.

#### 3.5.3. Co-Ordinated and Integrated Care

After adjusting for all co-variates, a high school education or below, no private health insurance, and not being depressed were associated with higher perceived quality of co-ordinated and integrated care. However, being employed was associated with lower perceived quality of co-ordinated and integrated care.

#### 3.5.4. Emotional Support

Compared to those diagnosed with leukemia, a lymphoma diagnosis or myeloma diagnosis was associated with higher perceived quality of emotional support as was not being depressed after adjusting for all co-variates. Not being anxious was associated with lower perceived quality of emotional support.

#### 3.5.5. Follow-Up Care

After adjusting for all co-variates, patients with no private health insurance and who were not depressed had higher perceived quality of follow-up care. Compared to those diagnosed >24 months ago, a diagnosis ≤12 months ago was associated with lower perceived quality of follow-up care.

#### 3.5.6. Respectful Communication

Perceived quality of respectful communication was higher among patients without depression compared to those with depression after adjusting for all co-variates.

#### 3.5.7. Cancer Information

Being female, a diagnosis of other hematological conditions (compared to leukemia), and attendance at treatment center 3 (compared to center 1) were associated with lower perceived quality of cancer information after adjusting for all co-variates. A diagnosis ≤12 months ago (compared to >24 months ago), no private health insurance, and not being depressed were associated with higher perceived quality of cancer information.

#### 3.5.8. Equitable Care

After adjusting for all co-variates, compared to those diagnosed with leukemia, a lymphoma diagnosis was associated with higher perceived equitable care.

None of the characteristics examined (i.e., age, sex, education, employment, marital status, private health insurance, location of residence, time since diagnosis, cancer type, depression, anxiety, and treatment center attended) were associated with the timely care, or patient preferences and values subscales.

## 4. Discussion

This study found that most patients perceived that hospital staff used *respectful communication* during interactions with hematological cancer patients. Specifically, the three most commonly delivered areas of patient-centered care related to hospital staff showing respect for the patient, showing respect for their family or friends, and speaking to the patient in a manner they could understand. This is similar to two prior studies that administered the QPCCC measure to hematological cancer survivors recruited via cancer registries [21] and medical oncology patients [27] in which participants rated the respectful communication items within the four most commonly delivered areas of patient-centered care. The consistency across the three studies suggests the potential generalizability of the results across treatment settings and cancer populations in Australia. Furthermore, our findings are consistent with evidence from other countries that most cancer patients believed that doctors and nurses were respectful to them [28,29], as well as their family and friends [29], and communicated information clearly [30].

Areas of care in which improvements were needed were also identified in the current study. A substantial proportion of hematological cancer patients perceived that hospital staff did not help them (30%) or their family/friends (29%) find others in a similar situation to talk to. These findings are similar to the findings of two previous studies, in which hematological cancer survivors rated these same items as the most common areas of care not delivered [21], while they were the second and third most common areas reported as not delivered by medical oncology patients [27]. A study conducted in Germany reported that 18% of patients with multiple myeloma wanted to use peer support groups [31]. While not all patients may wish to use this kind of support, it is still important that such care is routinely offered. Peer support programs for cancer patients have been found to increase knowledge about the condition and treatment and produce psychosocial benefits [32,33,34]. To overcome the privacy issues involved with staff directly linking patients and/or their families/friends with others with similar experiences, hospital staff could advise patients and their family or friends that cancer organizations such as the Myeloma Foundation of Australia offer peer support services.

Other research has examined whether the quality of patient-centered cancer care varies across medical oncology clinics [27]. Although this existing study [27] did include some participants with hematological cancers (6.8% of sample), the majority of participants were being treated for solid tumours. This is the first study to examine whether hematological cancer outpatients’ perceptions of quality of care vary across hematological cancer clinics. Results showed that compared to hematological cancer patients that attended treatment center 1, attendance at treatment center 3 was associated with lower perceived quality of cancer information. This is similar to research that reported that the quality of *cancer information* differed between medical oncology treatment centers [27]. However, the characteristics associated with the greatest number of quality subscale scores were depression (6 of 10 subscales) and the type of hematological cancer diagnosed (5 of 10 subscales). Similarly, prior research with hematological cancer survivors also found associations between depression and perceived quality of treatment decision-making, follow-up care, respectful communication and cancer information, and associations between hematological cancer type and perceived quality of treatment delivery and cancer information [21]. In an effort to improve the perceived quality of care among hematological cancer patients with depression or leukemia, hospital staff should ensure that sufficient information about treatment options and side-effects is provided to them, that adequate support (e.g., the involvement of family or social worker, time to consider options) is offered to assist with decision-making, and that patients are offered psychosocial support services if needed.

The strengths of this study included the use of a comprehensive measure that has been shown to have acceptable reliability and validity with hematological cancer survivors [20] and medical oncology patients [27]. The study limitations included that there were statistically significant differences between those who completed the second survey that contained the QPCCC measure and those who completed the first survey only in terms of age (*p* = 0.008), marital status (*p* = 0.003), and employment (*p* = 0.047), and this may limit the generalizability of the findings. Furthermore, all three treatment centers were located in metropolitan public hospitals, and therefore the generalizability to treatment centers in private hospitals and rural areas may be limited. The recruitment of more patients from center 3 may also have provided a more comprehensive assessment of the quality of care delivered at that center. Additionally, two-fifths of the sample was diagnosed with lymphoma, and therefore these results may be less generalizable to patients diagnosed with less common types of hematological cancers. Another limitation is that this study did not consider whether haematological cancer patients had psychological support and, if so, the type used; hence, such information cannot be used to inform the results.

## 5. Conclusions

Assessing patient-centered care delivered to hematological cancer patients using valid and reliable measures is essential for prioritizing areas for quality improvement. Our study suggests that the provision of co-ordinated and integrated care to hematological cancer patients could be improved. Strategies that hospital staff could adopt to increase the provision of coordinated and integrated care include reminders in health care recording systems to refer patients to cancer organizations that can assist, for example, hematological cancer patients to connect with others in a similar situation. Future studies could compare the delivery of patient-centered care in more than three hematological cancer outpatient treatment centers, including those in rural locations, to gain a better understanding of the quality of patient-centered care that hematological cancer patients receive.

## Figures and Tables

**Table 1:** Hematological cancer patients’ characteristics.

	Patients (*n* = 215)	
	*n* ^1^	%
**Gender**		
Male	124	57.7
Female	91	42.3
**Age (years)**		
Mean (SD)	62 (13)	
**Marital status**		
Married/living with partner	156	
Separated/divorced/widowed/never married	56	
**Education**		
High school or below	93	43.7
Trade/vocational	68	31.9
University	52	24.4
**Employment**		
Employed	77	36.0
Not in labour force or unemployed	137	64.0
**Residence**		
Urban	203	95.8
Rural	9	4.2
**Cancer type**		
Lymphoma	92	43.0
Leukemia	54	25.2
Myeloma	53	24.8
Other blood cancer	15	7.0
**Time since diagnosis**		
<12 months	59	27.6
12–24 months	41	19.2
>24 months	114	53.3
**Private health insurance**		
Yes	128	60.1
No	85	39.9
**Treatments received ^2^**		
Surgery	37	17.7
Chemotherapy	162	76.4
Radiation therapy/radiotherapy	49	23.0
Hormone treatment	5	2.3
Antibody treatment	17	8.0
Bone marrow	21	9.9
Stem cell transplant	40	18.8
Targeted therapies	20	9.4
**Treatment center**		
Center 1	116	54.5
Center 2	70	32.9
Center 3	27	12.7

^1^ Totals may not equal 215 due to missing data. ^2^ Participants could report multiple treatments.

**Table 2:** Comparison of (a) patients who completed first survey and non-consenters and (b) participants who completed the QPCCC measure in second survey and those who completed first survey only.

Characteristics	Completed First Survey (*n* = 289) ^1^	Non-Consenters (*n* = 42) ^1^	*p*-Value
**Age (years)**			
<55	82 (29%)	11 (29%)	0.97
55–74	148 (53%)	21 (55%)	
≥75	51 (18%)	6 (16%)	
**Gender**			
Male	163 (58%)	22 (61%)	0.72
Female	120 (42%)	14 (39%)	
**Characteristics**	**Participants Completed Second Survey with QPCCC Measure (*n* = 215) ^1^**	**Completed First Survey only (*n* = 74) ^1^**	***p*-Value**
**Gender**			
Male	124 (58%)	39 (57%)	1.00
Female	91 (42%)	29 (43%)	
**Age (years)**			
<55	57 (27%)	25 (37%)	0.008 *
55–74	123 (58%)	25 (37%)	
≥75	33 (15%)	18 (26%)	
**Education**			
High school or below	93 (44%)	34 (50%)	0.24
Vocational training	68 (32%)	24 (35%)	
University degree	52 (24%)	10 (15%)	
**Marital status**			
Married or partner	156 (74%)	36 (53%)	0.003 *
Never married, divorced, separated, widowed	56 (26%)	32 (47%)	
**Employment**			
Paid employment	77 (36%)	28 (41%)	0.047 *
Not in labour force	133 (62%)	35 (51%)	
Unemployed	4 (1.9%)	5 (7.4%)	
**Time since diagnosis**			
<12 months	59 (28%)	25 (37%)	0.31
12–24 months	41 (19%)	10 (15%)	
>24 months	114 (53%)	32 (48%)	
**Area of residence**			
Urban	203 (96%)	63 (97%)	1.00
Rural	9 (4.2%)	2 (3.1%)	
**Cancer type**			
Lymphoma	92 (43%)	30 (45%)	1.00
Leukaemia	54 (25%)	17 (25%)	
Myeloma	53 (25%)	16 (24%)	
Other blood cancer	15 (7.0%)	4 (6.0%)	

^1^ May not equal totals due to missing data. * *p* < 0.05.

**Table 3:** Cancer care most commonly delivered.

Item	Strongly Agree/Agree		QPCCC Subscale
	*n*	% ^1^ (95% CIs)	
The staff at the hospital showed respect for me	191	91.0 (87.1–94.8)	Respectful communication
The staff at the hospital talked to me in a way I could understand	188	90.8 (86.9–94.8)	Respectful communication
The staff at the hospital showed respect for my family or friends	183	87.6 (83.1–92.0)	Respectful communication
The staff at the hospital gave me information about cancer that was easy to understand	183	87.1 (82.6–91.7)	Cancer information
The staff at the hospital gave me information about cancer and treatments to take home (e.g., booklets, websites)	180	86.1 (81.4–90.8)	Cancer information
During my treatment, staff at the hospital made sure I received the treatment I was meant to have	182	85.8 (81.2–90.5)	Treatment delivery
The doctors at the hospital explained to me the short-term side effects of each treatment option	175	84.5 (79.6–89.5)	Treatment decision-making
The doctors at the hospital explained to me all of the treatments I could have	175	83.7 (78.7–88.7)	Treatment decision-making
During my treatment, staff at the hospital had up-to-date information about my cancer care	173	83.2 (78.1–88.3)	Treatment delivery
During my treatment, staff at the hospital made sure I received treatment that was based on scientific knowledge	169	82.0 (76.8–87.3)	Treatment delivery
During my treatment, staff at the hospital gave me consistent information about my treatment	169	80.9 (75.5–86.2)	Treatment delivery
During my treatment, staff at the hospital co-ordinated my appointments so that I did not have to go to hospital more than necessary	168	80.0 (74.6–85.4)	Treatment delivery

^1^ Denominators used to calculate percentages may differ due to missing data.

**Table 4:** Cancer care most commonly not delivered.

Item	Strongly Disagree/Disagree		QPCCC Subscale
	*n*	% ^1^ (95% CIs)	
The staff at the hospital helped me find other cancer patients I could talk to about their cancer experiences	61	29.8 (23.5–36.0)	Coordinated and integrated care
The staff at the hospital helped my family or friends find others in a similar situation to talk to	59	28.9 (22.7–35.1)	Coordinated and integrated care
The doctors at the hospital explained to me I could get a second medical opinion if I wanted to	55	26.6 (20.6–32.6)	Treatment decision-making
The doctors at the hospital explained to me how each treatment option might affect my length of life	47	22.8 (17.1–28.5)	Treatment decision-making
The staff at the hospital helped me get parking at the hospital that was affordable	44	21.4 (15.8–27.0)	Coordinated and integrated care
During my treatment, I was able to choose which doctor I saw for each appointment	44	21.0 (15.4–26.5)	Patient preferences and values

^1^ Denominators used to calculate percentages may differ due to missing data.

**Table 5:** QPCCC subscale scores.

QPCCC Subscale ^1^	*n* ^2^	Mean (SD)
Respectful communication	207	3.6 (0.6)
Treatment delivery	208	3.4 (0.6)
Cancer information	206	3.3 (0.7)
Treatment decision-making	208	3.2 (0.6)
Timely care	209	3.2 (0.8)
Follow-up care	208	3.0 (0.6)
Equitable care	209	3.0 (0.7)
Patient preferences and values	209	2.9 (0.8)
Emotional support	211	2.8 (0.5)
Coordinated and integrated care	206	2.5 (0.4)

^1^ 1 = lowest quality to 4 = highest quality. ^2^ Completed ≥70% of subscale items.

**Table 6:** Characteristics associated with patients’ perceptions of care.

	**Treatment Delivery**		**Treatment Decision-Making**		**Coordinated and Integrated Care**		**Emotional Support**		**Timely Care**	
	**Estimated Change (95% CI)**	***p***	**Estimated Change (95% CI)**	***p***	**Estimated Change (95% CI)**	***p***	**Estimated Change (95% CI)**	***p***	**Estimated Change (95% CI)**	***p***
**Treatment center attended**										
Center 1	ref	0.55	ref	0.75	ref	0.35	ref	0.70	ref	0.89
Center 2	−0.00 (−0.30, 0.30)		0.03 (−0.18, 0.25)		0.00 (−0.07, 0.07)		0.06 (−0.10, 0.22)		−0.04 (−0.40, 0.32)	
Center 3	−0.23 (−0.65, 0.20)		−0.09 (−0.38, 0.21)		−0.07 (−0.17, 0.03)		−0.01 (−0.24, 0.21)		−0.12 (−0.63, 0.38)	
**Education**										
High school or less	0.32 (0.03, 0.60)	0.03 *	0.25 (0.06, 0.45)	0.01 *	0.07 (0.01, 0.14)	0.03 *	0.06 (−0.09, 0.21)	0.41	−0.19 (−0.52, 0.15)	0.28
University/trade/vocational	ref		ref		ref		ref		ref	
**Marital status**										
Married or partner	0.00 (−0.31, 0.32)	0.98	0.07 (−0.15, 0.29)	0.53	0.07 (−0.00, 0.14)	0.05	0.08 (−0.09, 0.24)	0.37	−0.28 (−0.65, 0.09)	0.14
Never married, divorced, separated, or widowed	ref		ref		ref		ref		ref	
**Employment**										
Full or part time work	0.02 (−0.65, 0.69)	0.77	−0.21 (−0.68, 0.26)	0.31	−0.29 (−0.44,−0.13)	<0.001 *	−0.20 (−0.56, 0.16)	0.48	−0.52 (−1.33, 0.29)	0.43
Home duties, unemployed, retired, disabled	0.12 (−0.52, 0.76)		−0.31 (−0.76, 0.15)		−0.29 (−0.44, −0.14)		−0.21 (−0.56, 0.13)		−0.50 (−1.27, 0.27)	
Other	ref		ref		ref		ref		ref	
**Private health insurance**										
Yes	ref	0.04 *	ref	0.12	ref	0.04 *	ref	0.44	ref	0.95
No	0.32 (0.02, 0.61)		0.17 (−0.04, 0.37)		0.07 (0.00, 0.14)		0.06 (−0.10, 0.22)		−0.01 (−0.37, 0.35)	
**Sex**										
Male	ref	0.05	ref	0.08	ref	1.00	ref	1.00	ref	0.79
Female	−0.27 (−0.54, 0.00)		−0.17 (−0.36, 0.02)		−0.00 (−0.06, 0.06)		−0.00 (−0.14, 0.14)		0.04 (−0.28, 0.37)	
**Residence**										
Urban	0.17 (−0.51, 0.85)	0.62	0.12 (−0.36, 0.60)	0.63	−0.14 (−0.31, 0.03)	0.10	0.14 (−0.23, 0.50)	0.46	0.22 (−0.60, 1.03)	0.60
Rural	ref		ref		ref		ref		ref	
**Age**										
18–49 years	0.07 (−0.32, 0.46)	0.73	0.05 (−0.23, 0.32)	0.73	0.00 (−0.09, 0.09)	1.00	0.11 (−0.10, 0.32)	0.29	−0.24 (−0.71, 0.23)	0.31
50+ years	ref		ref		ref		ref		ref	
**Time since diagnosis**										
≤12 months	0.15 (−0.17, 0.48)	0.63	−0.13 (−0.36, 0.09)	0.38	0.00 (−0.08, 0.08)	1.00	0.01 (−0.16, 0.18)	0.76	−0.09 (−0.48, 0.29)	0.71
13–24 months	0.07 (−0.29, 0.43)		−0.14 (−0.39, 0.12)		−0.00 (−0.08, 0.08)		−0.06 (−0.25, 0.13)		0.11 (−0.33, 0.55)	
>24 months	ref		ref		ref		ref		ref	
**Cancer type**										
Lymphoma	0.56 (0.21, 0.91)	0.02 *	0.46 (0.21, 0.70)	0.002 *	−0.00 (−0.08, 0.08)	0.19	0.22 (0.04, 0.41)	0.04 *	0.18 (−0.23, 0.60)	0.62
Myeloma	0.33 (−0.05, 0.71)		0.21 (−0.06, 0.48)		0.07 (−0.02, 0.16)		0.25 (0.05, 0.45)		0.09 (−0.36, 0.54)	
Leukaemia	ref		ref		ref		ref		ref	
Other	0.33 (−0.16, 0.82)		0.14 (−0.21, 0.49)		0.07 (−0.04, 0.19)		0.06 (−0.20, 0.32)		0.38 (−0.22, 0.97)	
**Anxiety**										
Yes	ref	0.70	ref	0.03 *	ref	1.00	ref	<0.001 *	ref	0.33
No	0.07 (−0.27, 0.41)		−0.27 (−0.51, −0.03)		−0.00 (−0.08, 0.08)		−0.39 (−0.57, −0.21)		−0.20 (−0.61, 0.21)	
**Depression**										
Yes	ref	0.82	ref	<0.001 *	ref	0.001 *	ref	<0.001 *	ref	0.11
No	0.04 (−0.33, 0.42)		0.55 (0.29, 0.82)		0.14 (0.05, 0.23)		0.44 (0.24, 0.64)		0.37 (−0.08, 0.82)	
	**Follow-up Care**		**Respectful Communication**		**Patient Preferences and Values**		**Cancer Information**		**Equitable Care**	
	**Estimated Change (95% CI)**	***p***	**Estimated Change (95% CI)**	***p***	**Estimated Change (95% CI)**	***p***	**Estimated Change (95% CI)**	***p***	**Estimated Change (95% CI)**	***p***
**Treatment center attended**										
Center 1	ref	0.94	ref	1.00	ref	0.30	ref	0.001 *	ref	0.24
Center 2	−0.04 (−0.28, 0.21)		0.00 (−0.16, 0.16)		0.12 (−0.17, 0.42)		0.29 (0.05, 0.52)		0.09 (−0.16, 0.34)	
Center 3	0.02 (−0.33, 0.38)		0.00 (−0.23, 0.23)		0.32 (−0.10, 0.75)		−0.33 (−0.66, −0.00)		0.30 (−0.06, 0.65)	
**Education**										
High school or less	0.02 (−0.21, 0.25)	0.86	0.00 (−0.15, 0.15)	1.00	0.04 (−0.23, 0.32)	0.76	0.14 (−0.08, 0.36)	0.20	−0.20 (−0.44, 0.03)	0.09
University/trade/vocational	ref		ref		ref		ref		ref	
**Marital status**										
Married or partner	0.04 (−0.22, 0.29)	0.77	0.00 (−0.17, 0.17)	1.00	−0.04 (−0.35, 0.26)	0.78	0.19 (−0.05, 0.43)	0.12	0.05 (−0.21, 0.30)	0.72
Never married, divorced, separated, or widowed	ref		ref		ref		ref		ref	
**Employment**										
Full or part time work	−0.18 (−0.74, 0.37)	0.75	0.17 (−0.20, 0.53)	0.64	0.29 (−0.38, 0.95)	0.53	0.36 (−0.18, 0.91)	0.43	0.02 (−0.57, 0.61)	0.41
Home duties, unemployed, retired, disabled	−0.20 (−0.73, 0.33)		0.17 (−0.18, 0.52)		0.35 (−0.28, 0.99)		0.32 (−0.21, 0.84)		−0.14 (−0.71, 0.43)	
Other	ref		ref		ref		ref		ref	
**Private health insurance**										
Yes	ref	0.04 *	ref	1.00	ref	0.68	ref	0.005 *	ref	0.71
No	0.26 (0.01, 0.50)		0.00 (−0.16, 0.16)		0.06 (−0.23, 0.35)		0.33 (0.10, 0.57)		0.05 (−0.20, 0.29)	
**Sex**										
Male	ref	0.85	ref	1.00	ref	1.00	ref	0.009 *	ref	1.00
Female	0.02 (−0.20, 0.24)		−0.00 (−0.15, 0.15)		0.00 (−0.27, 0.27)		−0.29 (−0.50, −0.07)		−0.00 (−0.23, 0.23)	
**Residence**										
Urban	0.49 (−0.07, 1.05)	0.09	−0.00 (−0.37, 0.37)	1.00	0.26 (−0.41, 0.94)	0.44	−0.13 (−0.66, 0.40)	0.62	0.51 (−0.05, 1.08)	0.07
Rural	ref		ref		ref		ref		ref	
**Age**										
18–49 years	0.26 (−0.07, 0.58)	0.12	0.00 (−0.21, 0.21)	1.00	0.04 (−0.35, 0.42)	0.86	0.29 (−0.02, 0.59)	0.06	0.25 (−0.07, 0.57)	0.13
50+ years	ref		ref		ref		ref		ref	
**Time since diagnosis**										
≤12 months	−0.28 (−0.55, −0.01)	0.03 *	0.00 (−0.18, 0.18)	1.00	−0.02 (−0.34, 0.30)	0.98	0.48 (0.22, 0.73)	<0.001 *	−0.05 (−0.31, 0.22)	0.64
13–24 months	0.16 (−0.14, 0.45)		0.00 (−0.20, 0.20)		−0.04 (−0.39, 0.32)		0.29 (0.00, 0.57)		0.11 (−0.18, 0.41)	
>24 months	ref		ref		ref		ref		ref	
**Cancer type**										
Lymphoma	0.14 (−0.15, 0.43)	0.69	−0.00 (−0.19, 0.19)	0.05	0.44 (0.09, 0.78)	0.07	−0.14 (−0.42, 0.13)	0.02 *	0.41 (0.12, 0.70)	0.03 *
Myeloma	0.18 (−0.13, 0.49)		−0.00 (−0.21, 0.21)		0.24 (−0.13, 0.61)		−0.14 (−0.44, 0.15)		0.16 (−0.16, 0.47)	
Leukaemia	ref		ref		ref		ref		ref	
Other	0.13 (−0.28, 0.53)		−0.33 (−0.60, −0.06)		0.42 (−0.06, 0.90)		−0.62 (−1.00, −0.23)		0.09 (−0.31, 0.49)	
**Anxiety**										
Yes	ref	0.75	ref	1.00	ref	0.64	ref	1.00	ref	1.00
No	−0.05 (−0.33, 0.23)		0.00 (−0.19, 0.19)		−0.08 (−0.42, 0.26)		0.00 (−0.27, 0.27)		−0.00 (−0.28, 0.28)	
**Depression**										
Yes	ref	0.03 *	ref	0.001 *	ref	0.46	ref	<0.001 *	ref	0.11
No	0.35 (0.04, 0.66)		0.33 (0.13, 0.54)		0.14 (−0.23, 0.51)		0.57 (0.27, 0.87)		−0.25 (−0.56, 0.06)	

* *p* < 0.05.
